# Multielemental Analysis and *In Vitro* Evaluation of Free Radical Scavenging Activity of Natural Phytopigments by ICP-OES and HPTLC

**DOI:** 10.3389/fphar.2021.620996

**Published:** 2021-07-06

**Authors:** S. M. Nandanwadkar, P. J. Hurkadale, C. M. Bidikar, M. M. Godbole

**Affiliations:** KLE Academy of Higher Education and Research, Belgaum, India

**Keywords:** phytopigments, ethnomedicinal plants, ICP-OES, complete digestion, DPPH-HPTLC

## Abstract

The phytopigments derived from ethnomedicinal plants employed as traditional medicines appear to be the simplest alternative for artificial radical colorants. This can be because of persistent use of synthetic dyes and their harmful impacts linked to human lives as well as to the ecosystem. The literature evidences clearly reveal the complications from growing demands of radical colorants from artificial origin. The planned analysis work hence focuses on screening of the fundamental composition of phytopigments, obtained from plant sources by subtle technique of ICP-OES, with axial plasma combined with nebulizer motor–assisted gas flow approach, utilizing microwave digester for complete digestion of phytopigments, thereby establishing the pigments being safe for consumption. Additionally, the observations from free radical scavenging activity using DPPH by HPTLC concluded that the natural pigments obtained from plant sources are rich in flavonoids with potent antioxidant property. Thus, an effort has been made through the developed ICP-OES methodology, to beat the distinct imprecise practice of food labeling, once natural pigments are utilized in a variety of additives, as food colorants with amounts of components detected as arsenic, lead, and metal, within specified limits of FSSAI, demonstrate and establish safety of natural foodstuff agents, as compared over hazardous synthetic azo dyes.

## Introduction

Colors form an integral component of food and related consumer goods because of their intrinsic indispensable fundamental property of imparting unique characteristic hues to the product to which they are added. These food colors not only define nature of food but also are linked to human desires for types of food consumed; for example, yellow to light green for raw fruits with citrus flavor (acidic), whereas red and pink for ripe condition with sweet taste. Routine food colors are classified into tetrapyrroles, tetraterpenoids, flavonoids, *etc*. Some class of pigments were also further categorized into anthocyanins (from blue berries), betalains (from beetroot), and annatto (from bixin seeds).

On the basis of their source of origin, colors may be classified as follows:

1. Natural: bixin, anthocyanins, betanins, chlorophylls, curcumin, lycopene, *etc*.
2. Nature identical: orange, watermelon, *etc*.
3. Artificial colors: blue-1, blue-2, tartrazine, erythrosine, citrus red-2, indigo carmine, Ponceau 4R, *etc*.
4. Inorganic: TiO_2_ (from metals) (Mortensen, 2006).

The recent advancements in technology for extraction and assessment of biological activities like antiaging phenomena as well as therapeutic efficacy like treating blood disorders, anemia, beneficial effect on lungs, free radical scavenging activity, and the newer phytopigments like anthocyanins, betanins, curcumins, indigotin, and isatin are the preferred choice of coloring agents in foods by public considering focal point of their safety as well as for manufacturers in regards to expanding their extensive business (Graham et al., 1990; Micozzi et al., 1990; Zhang et al., 1991; Sies et al., 1992; Van Poppel, 1993). In terms of food frauds, the establishment of accurate and rapid instrumental techniques for natural colorings is required, especially for the phytopigments like anthocyanins and betalains; oleo-resins like paprika and turmeric; carotenoids; and quinoids, that are off late frequently utilized in different foods and related consumer matrices (Fujii et al., 2001). Frugal, fast, and concurrent analytical techniques need to be established. Elemental estimation is another parameter of great significance for determining beneficial and toxic effects of plant pigments, like anthocyanin, betanin, paprika oleoresin, annatto, and turmeric oleoresin, that contain adequate basic components additionally for human well-being (Kohen and Nyska, 2002). Most recent innovations in Foodomics as well as farsighted toxicological studies have been applied for screening potential hazardous synthetic contaminants such as heavy metals and aflatoxins in early medication improvement/new drug lead molecule discoveries. Hence, such novel innovations would surely be utilized to add to the scientific evidence to plant pigments in regards to its safety (Jordan et al., 2010). The overall expanding enthusiasm for natural cycles just as the synthetic structure of plants for dietary, restorative, and ecological issues has led to advanced metal-related studies (Adriano, 2001). Recent dossiers and newer regulations regarding stringent food norms and laws prompt more noteworthy entry of toxic metals into the climate. with the indication that a critical aspect of the populace is currently routinely exposed to these toxic metals (Goyer, 1997). Plants are probable sources for micro- and macro-essential nutrients, since there are different take-up courses for fundamental components such as uptake of minerals, salts, and metals from soil (Barthwal et al., 2008). These components are utilized, for instance, keeping up osmotic balance, as basic parts in sugars and proteins, as segments of natural atoms associated with typical digestion (e.g., magnesium (Mg) in chlorophyll, phosphorous (P) in ATP, iron (Fe) in photosynthesis, copper (Cu) in root digestion, and zinc (Zn) for development), and as chemical activators (e.g., potassium (K) in tissue cells and layers, calcium (Ca) for stress reaction, (K) as development controller, and nickel (Ni); Hepler, 2005; Tanhan et al., 2007; Soetan et al., 2010; Wang et al., 2013). Adjacent to the fundamental components, a few metals, for example, arsenic (As), cadmium (Cd), and lead (Pb), are toxic to the creature even in exceptionally low levels, while other do not show any positive or negative impact. It is vital, particularly for spices, that they contain adequate fundamental components (micro–macro nutrients) additionally for human well-being. Besides, micro-components in plants are associated with the arrangement cycles of dynamic synthetic elements, which permit their utilization for treating various disorders, for example, iron (Fe) supplement for blood-related disorders. The detrimental impact results from the reality that heavy metals migrate from soils, harvests, or plants, entering the food chain (Antonijević et al., 2012), or by therapeutic applications from medicaments (Cheng, 1955; Garcia et al., 2000; Başgel and Erdemoğlu, 2006; Adeyolanu et al., 2016). Considering the aforementioned points, the World Health Organization (WHO) has specified the permissible levels in crude plant material for the presence of heavy metals, thereby maintaining a stringent watch of negative effects on human well-being by plant utilization or its clinical use (WHO, 1991; Zeiner et al., 2015).

Antioxidant assays might depend on the probable type of anticancer agents (lipophilic or hydrophilic, and enzymatic or nonenzymatic) (Huang et al., 2005). The DPPH test by HPTLC is one of the reliable mainstreams and frequently preferred technique among diagnostic tools for identifying the presence of radical scavenging/antioxidant activity. The technique is basic, productive, moderately reasonable, and fast. The shading change occurs from purple color of the reagent to lemon yellow color components possessing free radical scavenging property, permitting the spectrophotometric assurance of the agent possessing possible free radical scavenging (antioxidant) activity (Molyneux, 2004; Kedare and Singh, 2011).

In the proposed research, an effort has been made to feature the biotherapeutic potential of phytopigments utilized in foods and related food merchandises, by chromatographic fingerprints *via* free radical activity, along with multielemental assessment using inductive coupled plasma-optical emission spectroscopy (ICP-OES), with a viewpoint to screen heavy metals such as lead, arsenic, and cadmium, in addition to micro- and macro-minerals like Mg, Fe, and Zn. Therefore, the novel strategy developed above would surely be employed in routine quality control for monitoring food.

## Experimental

### Reagents

Natural pigments like anthocyanins, betanins, curcumin oleoresin, paprika oleoresin, and annatto were procured from Neelikon Food Dyes Pvt. Ltd., Mumbai. 98% nitric acid-reagent grade (Fisher Scientific, Fair Lawn, NJ, United States). Hydrogen peroxide (H_2_O_2_)-30% reagent grade (J.T. Baker, Phillipsburg, NJ, United States). Micro laboratory cleaner (International Products Corporation, Trenton, NJ, United States. Distilled, deionized water, 18 MΩ (Continental Water Systems, San Antonio, TX, United States). For HPTLC analysis, n-butanol, glacial acetic acid, water, and DPPH (1,1-diphenyl-2-picrylhydrazyl) were of analytical grade and purchased from Merck and Sigma Chemical, Germany.

### Apparatus

ICP AVIO 200 inductively coupled plasma optical emission spectrometer was purchased from Perkin-Elmer, Ahmedabad, Gujarat. Titan MPS microwave digestor. Teflon® PFA microwave vessels rated to 120 psi (CEM, Matthews, NC, United States). Lined digestion vessels rated to 200 psi (CEM, Matthews, NC, United States). Food processor was designed for home use. High-density polyethylene bottles, Nalgene®, or equivalent were purchased from Nalge Company, Rochester, NY, United States. The HPTLC system purchased from Camag was used for TLC analysis, and data interpretation was performed using digitally optimized vision CATS Software. Camag derivatizer was employed for derivatization to demonstrate free radical antioxidant activity using DPPH.

### Instrumentation

A Titan MPS microwave digestor 950 W power with temperature and pressure monitoring and control was utilized to digest all the samples. The microwave was aligned after the U.S. Environmental Protection Agency (EPA) calibration procedure that involves heating a known volume of water at specific powers (Hepler, 2005). Individual experience directs that the sample weights should be kept at or below the prescribed weights to minimize the potential for overpressuring and venting during the microwave digestion process. Samples containing high levels of sugars are apt to react more quickly and to generate more pressure. If these products are substituted for the materials analyzed in this work, using smaller sample sizes and lower powers are strongly recommended to prevent overpressuring.

The ICP-OES analysis was performed on the Perkin-Elmer ICP AVIO 200 inductively coupled plasma-optical emission spectrometer equipped with an axial standard torch using cross-flow nebulizer. Autosampler featuring a quartz sample probe to minimize sample cross contamination was used. The working specifications of ICP-OES Avio 200 and optimal conditions of plasma are given in Table 1. ICP AVIO 200 was used because of its extremely sensitive precision to detect metals at trace levels. Also, due to the simultaneous measurement, no reduction in sample throughput was observed from making measurements at multiple wavelengths of an element. Therefore, multiple emission lines were measured simultaneously for each element to verify analytical results. The analysis of different types of the samples of phytopigments was performed on different days and because each sample was in a different acid matrix, optimum background correction points (BGC) and peak windows were determined as a function of sample before the analysis was performed. The standard concentrations were matched to the anticipated levels in the diluted samples (Fikarová, 2020). The Camag HPTLC system was employed for chromatographic analysis.

**TABLE 1:** Plasma specification and optimum conditions for ICP-OES.

Optimum conditions	Parameters
Plasma power	1,350–1,500 w
Gas flow	0.2 (nebulizer)/0.8 auxiliary (liters/minutes)
Coolant	15°C (55°F)
Nebulizer type	Cross flow
Nebulizer flow rate	0.2–0.8 (liters/minutes)
Pump speed	6.2 RPM
Stabilization time	60 s
No. of probes for each measuring	3
Plasma observation	Axial (low concentration), radial (high concentration)

### Sample Pretreatment for Inductive Coupled Plasma-Optical Emission Spectroscopy Analysis Using Avio 200 Perkin Elmer System

The phytopigments obtained from Neelikon Pvt Ltd. were pretreated using digestion mixture of concentrated nitric acid (HNO_3_) and sulfuric acid (H_2_SO_4_), utilizing the Perkin Elmer Titan MPS microwave digestor (Liu et al., 2020).

### Digestion Procedure for Inductive Coupled Plasma-Optical Emission Spectroscopy Analysis

Step 1: 10 gm of each selected phytopigments were accurately weighed into the Teflon PFA digestion vessels.
Step 2: Concentrated, ultrapure HNO_3_ and 2 mL concentrated, ultrapure H_2_SO_4_ were added to the sample.
Step 3: Cap the vessel in the capping station.
Step 4: Phytopigment powders were digested as per Table 2.
Step 5: Cool for approximately five minutes and vent vessels.
Step 6: Repeat Step 4 for juices containing pulp.
Step 7: Cool for five minutes, vent, and open the vessels using the capping station.
Step 8: Add 3 mL 30% H_2_O_2_ to the samples.
Step 9: After effervescence subsides, transfer the samples into clean, acid-washed volumetric flasks, and dilute to 100 mL with double distilled water.
Step 10: Transfer the samples to clean HDPE bottles.

**TABLE 2:** Microwave digestion parameters for phytopigment analysis.

Parameter	Stage 1	Stage 2	Stage 3	Stage 4	Stage 5
Power (%)	10	45	45	45	45
Power (watts)	51	366	366	366	366
Pressure (PSI)	20	40	80	120	160
Run time (min)	2	10	10	10	20
Time at parameter (min)	2	5	5	5	10
Temperature at parameter (ºC)	0	75	85	100	120
Fan speed (% of maximum)	100	100	100	100	100

### Standard Preparation for HPTLC Analysis

Standard solutions of selected phytopigments (anthocyanins, betanins, turmeric oleoresin, paprika oleoresin, and annatto) were of 100 ppm using methanol.

### Sample Preparation for HPTLC Analysis

Marketed food samples such as locally available jams, squashes, ice cream flavors, and chili powders, containing anthocyanins, betanins, paprika oleoresin, and annatto, were accurately weighed as per requirement (100 ppm) and mixed with a mixture of methanol and water (8:2 v/v). For turmeric samples, locally available turmeric powder was obtained from the market, and 10 ppm solution was prepared and further subjected to spectroscopic and chromatographic analyses by ICP-OES and HPTLC techniques.

### Optimized Conditions for HPTLC

Chromatography was performed on 20 × 10 cm aluminum-coated silica gel 60 F_254_ HPTLC plates (C.A.S. No. 105642, Merck, Darmstadt, Germany). Sample application was done using Camag Linomat V with 100 µL (Hamilton syringe, United States). Development was done using a solvent system of n-butanol: glacial acetic acid: water (4:4:1 v/v/v) up to 70 mm in twin-trough chamber with saturation of 10 min with 5 min plate equilibrium. Spectrodensitometric scanning was performed post-derivatization using the DPPH reagent at 540 nm.

### 1,1-Diphenyl-2-picrylhydrazyl Radical Scavenging

After development, the plates were dried for 10 min using a Camag plate heater III. Derivatization was done using 0.5 mM arrangement of 1,1-diphenyl-2-picrylhydrazyl (DPPH) as the reagent in ethanol for 5 s (Anuyahong et al., 2020). HPTLC plates were dried at room temperature (23°C) for 90 s and then warmed for 30 s at 60°C in a dark room. Densitometric scanning was performed at 540 nm using a Camag TLC scanner four under programming control of vision CATS, v 2.5 Muttenz, Switzerland. Zones were recognized promptly as lemon yellow regions against light violet/purple zones (bands). Estimations were done in duplicate. Ascorbic acid was utilized as a positive control.

## Results and Discussion

For heavy metal analysis of phytopigments, nine references of heavy metal standards, Ba, Cd, Cr, Co, Cu, Pb, Ni, As, and Se, were calibrated for analysis of anthocyanins, betanins, and paprika oleoresin, from which toxic heavy metals, when subjected to ICP-OES analysis, particularly, Pb, As, and Cd, were found to be well below the specified limits of the U.S. FDA and FSSAI regulations. The concentrations of As, Cd, Pb, and Hg were under the detection limit in each sample, which means that the investigated phytopigment samples of interest were free of toxic metals (Table 3). On a beneficial note, the nutritive profile of frequently used phytopigments was determined. Several studies have shown that the peel of beetroot was characterized by the largest betalain content (Kujala et al., 2002; Shing and Hathan, 2014; Slatnar et al., 2015; Sawicki et al., 2016). In this study, test sample of blue berry squashes and jams demonstrated anthocyanins with the highest content of iron (20.52 mg/L), and beetroot juices demonstrated betanins with the highest quantity of magnesium (17.85 mg/L), along with considerable amounts of iron (5.097 mg/L) and calcium (5.368 mg/L). Locally marketed turmeric samples reported turmeric oleoresins with the highest concentration of magnesium (19.40 mg/L) followed by the iron content of 4.942 mg/L, whereas chili powders demonstrated paprika oleoresin, reporting the calcium content of 5.958 mg/L. Element composition with respect to macro- and microelemental concentrations of different phytopigment parts is shown in Tables 4, 5. The results in the proposed study indicate that multielemental estimation of natural pigments was successfully performed by the ICP-OES technique, proving it to be a preferred and precise tool in routine quality control of food and related merchandized goods, ensuring and assuring safety profile of food additives and contaminants in the form of heavy metals, if any.

**TABLE 3:** Quantification of heavy metals observed in selected phytopigments with prescribed limits.

		Heavy metals			
Sl. no	Name of the color	Arsenic (mg/kg) ≤ 1	Lead (mg/kg) ≤ 1	Cadmium (mg/kg) ≤ 1	Mercury (mg/kg) ≤ 2
1	Anthocyanins	0.11	0.103	0.043	<0.1
2	Paprika	0.014	0.120	0.042	0.013
3	Betanin	0.022	0.167	0.045	0.014
4	Turmeric	–	0.061	0.049	–
5	Annatto	–	2.864	0.033	–

**TABLE 4:** ICP-OES results for selected phytopigments with reference values in mg/mL.

Phytopigment	Annatto	Anthocyanin	Betanins	Paprika	Turmeric
Minerals in highest quantity with observed amounts	Iron (Fe) 6.345 ± 0.40 mg/L, calcium (Ca) 4.868, and magnesium (Mg) 2.478	Iron (Fe) 20.58 ± 0.40 mg/L	Magnesium (Mg) 17.85 ± 0.4 mg/L with iron (Fe) 5.097 ± 0.40 mg/L and calcium (Ca) 5.368 ± 0.7 mg/L	Calcium (Ca) 5.958 ± 0.7 mg/L, iron (Fe) 0.278, and magnesium (Mg) 0.064 mg/L	Magnesium (Mg) 19.40 ± 0.40 mg/L and iron (Fe) 4.942 ± 0.40 mg/L mg/L

**TABLE 5:** Elemental analysis of phytopigment analysis with respective wavelengths and observed experimental values by ICP-OES AVIO 200 with reference values in mg/mL.

Element	Wavelength	Experimental	Label
Ca	396.845	51.3 ± 0.7	<115
Cu	224.702	0.137 ± 0.03	
Fe	238.200	0.59 ± 0.40	<2.1
K	766.515	0.681 ± 7.3	
Mg	279.553	42.70 ± 0.4	
Na	589.589	24.79 ± 0.02	57.5
P	177.436	139.51 ± 1.3	
Zn	206.198	0.79 ± 0.2	

**FIGURE 1:**
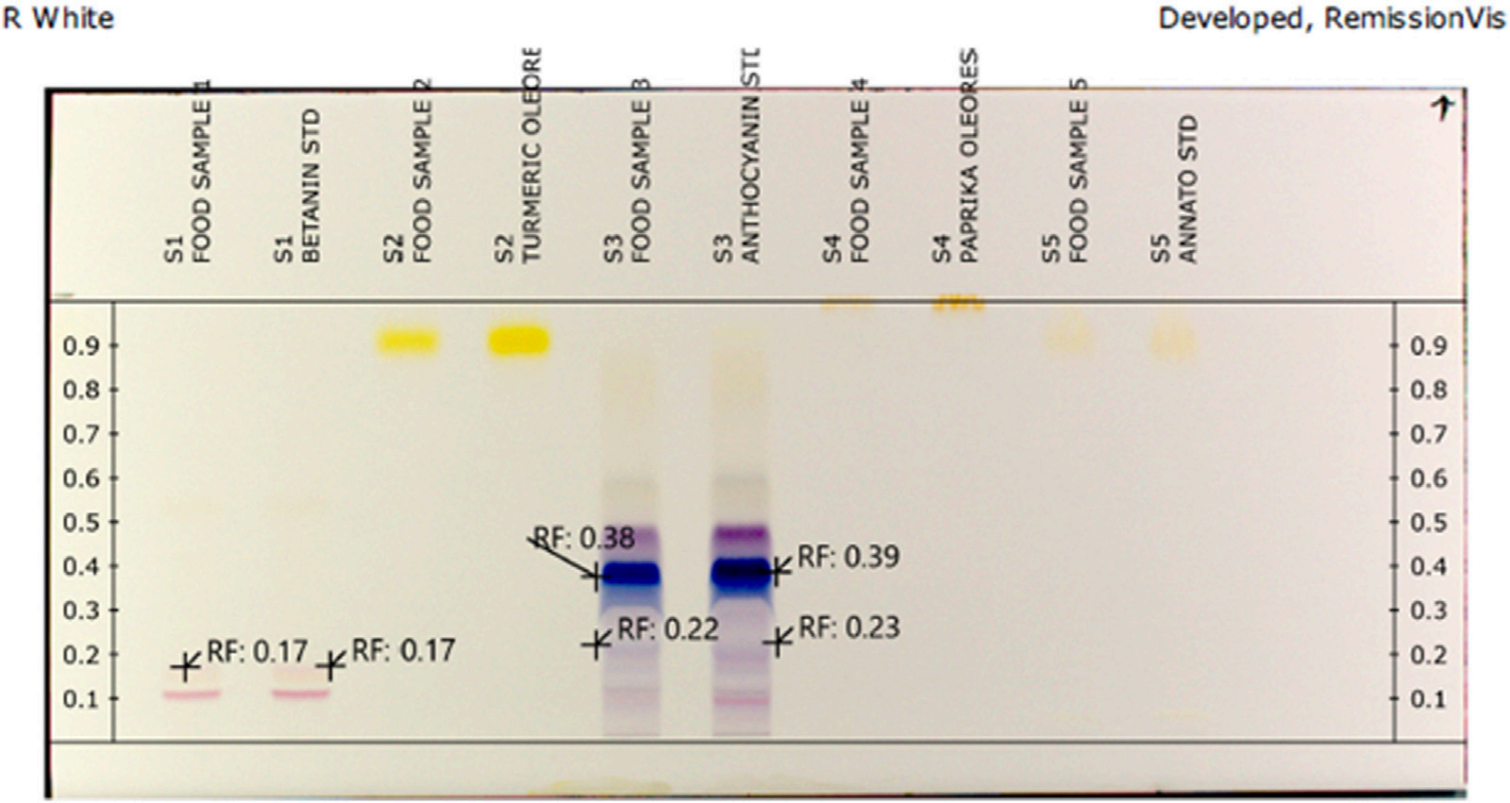
HPTLC fingerprint of selected phytopigments exhibiting antioxidant property post-derivatization by the DPPH reagent.

The utilization of HPTLC and a sophisticated scanner with a photodiode detector makes possible utilization of selected phytopigments on TLC plates without prior preparation steps (Pozharitskaya et al., 2007a; Pozharitskaya et al., 2007b). In a similar fashion, chromatographic separation, *in situ* radical scavenging activity examination of selected phytopigments was done by online HPTLC-DAD and HPTLC-DPPH techniques simultaneously. In this study, a simple and rapid HPTLC densitometric method for the analysis of selected phytopigments was developed. Different trial compositions of the solvent systems were put to task in order to obtain high-resolution and reproducible peaks. From preliminary experiments, the best results were obtained using the mobile phase of n-butanol: glacial acetic acid: water (4:4:1, v/v/v). Selected phytopigment samples were applied directly onto the plates without any prechromatographic separations. The DPPH reagent has a unique absorption maximum in the range of 510 and 520 nm, which is diminished in the presence of a compound capable of reducing it to its hydrazine form. The kinetic behavior of antioxidants is a significant factor in the assessment of antiradical activity. A reaction time of 30 s at 50°C was seen as the optimum antioxidant-DPPH response period. After chromatographic separation, *in situ* DPPH radical scavenging properties of phytopigments were controlled by an online HPTLC-DPPH technique. This is viewed as in Figure 1, by derivatized image of silica plates with DPPH radicals reducing light violet/purple bands to lemon yellow bands. These zones demonstrated antioxidant activity. The intensity of the lemon yellow color depended upon the amount and nature of radical scavenging activity in the sample.

## Discussion

The phytopigments were subjected to elemental estimation by the ICP-OES (AVIO-200 model) technique in accordance with pharmacopeial dossiers USP 231 and USP 233. The elemental characterization of selected natural pigments utilizing the Perkin Elmer TITAN MPS digestor equipped with nebulizer and dual axial plasma assisted fast elemental analysis. The developed protocol was validated by performing calibration using heavy metal reference standards of mercury (Hg), lead (Pb), cadmium (Cd), arsenic (As), selenium (Se), chromium (Cr), copper (Cu), cobalt (Co), and antimony (Sb). The ICP-OES method designed for elemental analysis gave accurate and precise nutritional profile for selected phytopigments in regards to inherent micro–macro essential elements like iron (Fe), calcium (Ca), zinc (Zn), and phosphorus (P). The ICP-OES outcome determined that heavy metals like arsenic (As), cadmium (Cd), lead (Pb), and mercury (Hg) were reported ≤1 ppm as specified within herbal pharmacopeia, labeling the selected phytopigments nonhazardous and safe for human consumption. The selected phytopigments reported essential minerals as follows: anthocyanins pigment reported the highest iron (Fe) content of 20.52 mg/L, and betanin pigment reported the highest magnesium (Mg) content of 17.85 mg/L, along with considerable amounts of iron (Fe) and calcium (Ca), of 5.097 mg/L and 5.368 mg/L, respectively; paprika oleoresin pigment reported only calcium (Ca) content of 5.958 mg/L. USP compliant DPPH assisted HPTLC protocol, developed for selected phytopigments, indicated that lemon yellow bands of phytopigments possess radical scavenging property with inherent chemical structure proving them to be antioxidant agents, suggesting them as future “Nutraceuticals.”

## Conclusion

The microwave-assisted digestion using the Perkin Elmer Titan MPS digestor gave a contamination-free sampling with minimal overall analysis time, providing faster, simpler, effective, and accurate estimation of elements. Toxic heavy metals, when subjected to the ICP-OES analysis, were within the specified limits of U.S. FDA and FSSAI regulations. On a beneficial note, the essential nutritive profile of frequently used phytopigments was determined. Annatto from butter had highest amounts of iron (Fe: 6.345 mg/L) , calcium (Ca: 5.368 mg/L), and magnesium content (2.478 mg/L). Anthocyanins had the highest content of iron (Fe: 20.52 mg/L) . Betanins had the highest quantity of magnesium (Mg: 17.85 mg/L) along with considerable amounts of iron (Fe: 5.097 mg/L) and calcium (Ca: 5.368 mg/L), with paprika oleoresin reporting calcium (Ca) content of 5.958 mg/L, whereas turmeric oleoresin from locally purchased turmeric powder demonstrated the highest content of magnesium (Mg: 19.40 mg/L), followed by the iron (Fe) content of 4.942 mg/L. The lemon yellow florescent bands indicated the presence of antioxidant property of the pigment.

The results in the proposed study indicate that multielemental estimation of phytopigments was successfully performed by the ICP-OES technique, proving it to be a preferred and precise tool as a routine quality control method ensuring and assuring safety profile of food additives and contaminants in the form of heavy metals, if any. The effect directed, antioxidant activity of the phytopigments was proven by 2,2-diphenyl-1-picrylhydrazyl (DPPH) test, using the CAMAG HPTLC system, where DPPH was used as a derivatizing reagent.

This work portrays the screening and identification of essential nutrients like iron, calcium, and magnesium as well as heavy metals in accordance with guidelines of FSSAI for foods (food industries) may be performed simultaneously utilizing the ICP-OES technique. The radical analysis carried out by the HPTLC-DPPH assay demonstrated possible inherent antioxidant properties of selected phytopigments.

## Data Availability

The original contributions presented in the study are included in the article/Supplementary Material; further inquiries can be directed to the corresponding author.
