# Supplementary material for: Multielemental Analysis and In Vitro Evaluation of Free Radical Scavenging Activity of Natural Phytopigments by ICP-OES and HPTLC
Source: Front Pharmacol. 2021 Jul 6;12:620996. doi: 10.3389/fphar.2021.620996 (PMC8290885; doi:10.3389/fphar.2021.620996)
Supplement: Supplementary file 2 [file DataSheet2.docx]

Table and table Caption

| **Optimum Conditions** | **Parameters** |
| --- | --- |
| Plasma power | 1350- 1500 Watts |
| Gas flow | 0.2(Nebulizer)/0.8 Auxiliary (Liters/Minutes) |
| Coolant | 15 º C (55 º F) |
| Nebulizer type | Cross flow |
| Nebulizer flow rate | 0.2 – 0.8 (Liters/Minutes) |
| Pump Speed | 6.2 RPM |
| Stabilization Time | 60 Seconds |
| No. of probes for each Measuring | 3 |
| Plasma observation | Axial (Low Concentration), Radial (High Concentration) |

**Table 1:** Plasma Specification and Optimum conditions for ICP-OES Avio 200

| **Parameter** | **Stage 1** | **Stage 2** | **Stage 3** | **Stage 4** | **Stage 5** |
| --- | --- | --- | --- | --- | --- |
| **Power (%)** | 10 | 45 | 45 | 45 | 45 |
| **Power (watts)** | 51 | 366 | 366 | 366 | 366 |
| **Pressure (PSI)** | 20 | 40 | 80 | 120 | 160 |
| **Run time (min)** | 2 | 10 | 10 | 10 | 20 |
| **Time at parameter (min)** | 2 | 5 | 5 | 5 | 10 |
| **Temperature at parameter (ºC)** | 0 | 75 | 85 | 100 | 120 |
| **Fan speed (% of maximum)** | 100 | 100 | 100 | 100 | 100 |

**Table 2:** Microwave Digestion Parameters for Phyto-pigment Analysis

|  | | ***HEAVY METALS*** | | | |
| --- | --- | --- | --- | --- | --- |
| **SL. No** | **Name of color** | **Arsenic (mg/Kg)≤ 1** | **Lead(mg/Kg) ≤ 1** | **Cadmium**  **(mg/Kg)≤ 1** | **Mercury**  **(mg/Kg)≤ 2** |
| **1** | **Anthocyanins** | 0.11 | 0.103 | 0.043 | < 0.1 |
| **2** | **Paprika** | 0.014 | 0.120 | 0.042 | 0.013 |
| **3** | **Betanin** | 0.022 | 0.167 | 0.045 | 0.014 |
| **4** | **Turmeric** | - | 0.061 | 0.049 | - |
| **5** | **Annatto** | - | 2.864 | 0.033 | - |

| **Phyto-pigments** | **Annatto** | **Anthocyanin** | **Betanins** | **Paprika** | **Turmeric** |
| --- | --- | --- | --- | --- | --- |
| **Minerals in highest quantity with observed amounts** | Iron (Fe) 6.345 ± 0.40 mg/L, Calcium (Ca) 4.868 and Magnesium (Mg) 2.478 | Iron (Fe) 20.58 ± 0.40 mg/L | Magnesium (Mg) 17.85 ± 0.4 mg/L with Iron (Fe) 5.097 ± 0.40 mg/L & Calcium (Ca) 5.368 ± 0.7 mg/L | Calcium (Ca) 5.958 ± 0.7 mg/L, Iron (Fe) 0.278 and Magnesium (Mg)0.064mg/L | Magnesium (Mg) 19.40± 0.40 mg/L and Iron (Fe) 4.942± 0.40 mg/L mg/L |

**Table3:** Quantification of Heavy metals observed in selected phyto-pigments with prescribed limits

**Table 4:** ICP-OES Results for selected Phyto-pigments with reference values in mg/mL

| **Element** | **wavelength** | **Experimental** | **Label** |
| --- | --- | --- | --- |
| **Ca** | 396.845 | 51.3± 0.7 | <115 |
| **Cu** | 224.702 | 0.137± 0.03 |  |
| **Fe** | 238.200 | 0.59± 0.40 | <2.1 |
| **K** | 766.515 | 0.681± 7.3 |  |
| **Mg** | 279.553 | 42.70± 0.4 |  |
| **Na** | 589.589 | 24.79± 0.02 | 57.5 |
| **P** | 177.436 | 139.51± 1.3 |  |
| **Zn** | 206.198 | 0.79± 0.2 |  |

**Table 5:** Elemental Analysis of Phyto-pigments analysis with respective wavelengths and observed experimental values by ICP-OES AVIO 200 with reference values in mg/mL
