# Supplementary figures and images for: Multielemental Analysis and In Vitro Evaluation of Free Radical Scavenging Activity of Natural Phytopigments by ICP-OES and HPTLC
Source: Front Pharmacol. 2021 Jul 6;12:620996. doi: 10.3389/fphar.2021.620996 (PMC8290885; doi:10.3389/fphar.2021.620996)

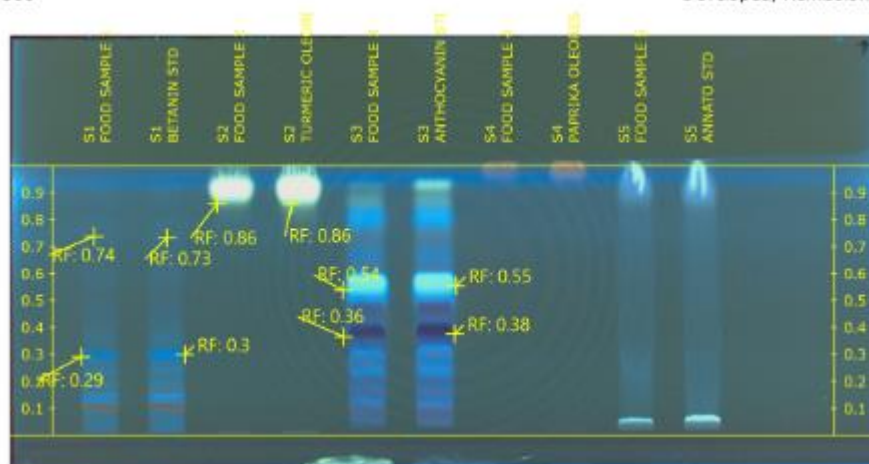

DPPH DERIVATIZED PLATE WITH LEMON YELLOW SPOTS / ZONES

Supplement: Supplementary file 4 [file Image1.pdf]
